# Intrathecal long-term gene expression by self-complementary adeno-associated virus type 1 suitable for chronic pain studies in rats

**DOI:** 10.1186/1744-8069-2-4

**Published:** 2006-01-30

**Authors:** Benjamin Storek, Nina M Harder, Michaela S Banck, Cheng Wang, Douglas M McCarty, William GM Janssen, John H Morrison, Christopher E Walsh, Andreas S Beutler

**Affiliations:** 1 Department of Medicine (Hematology/Oncology), Mount Sinai School of Medicine, New York, NY, USA; 2 Department of Neurosciences, Mount Sinai School of Medicine, New York, NY, USA; 3 Gene Therapy Center, University of North Carolina, Chapel Hill, NC, USA

## Abstract

**Background:**

Intrathecal (IT) gene transfer is an attractive approach for targeting spinal mechanisms of nociception but the duration of gene expression achieved by reported methods is short (up to two weeks) impairing their utility in the chronic pain setting. The overall goal of this study was to develop IT gene transfer yielding true long-term transgene expression defined as ≥ 3 mo following a single vector administration. We defined "IT" administration as atraumatic injection into the lumbar cerebrospinal fluid (CSF) modeling a lumbar puncture. Our studies focused on recombinant adeno-associated virus (rAAV), one of the most promising vector types for clinical use.

**Results:**

Conventional single stranded rAAV2 vectors performed poorly after IT delivery in rats. Pseudotyping of rAAV with capsids of serotypes 1, 3, and 5 was tested alone or in combination with a modification of the inverted terminal repeat. The former alters vector tropism and the latter allows packaging of self-complementary rAAV (sc-rAAV) vectors. Combining both types of modification led to the identification of sc-rAAV2/l as a vector that performed superiorly in the IT space. IT delivery of 3 × 10e9 sc-rAAV2/l particles per animal led to stable expression of enhanced green fluorescent protein (EGFP) for ≥ 3 mo detectable by Western blotting, quantitative PCR, and in a blinded study by confocal microscopy. Expression was strongest in the cauda equina and the lower sections of the spinal cord and only minimal in the forebrain. Microscopic examination of the SC fixed in situ with intact nerve roots and meninges revealed strong EGFP fluorescence in the nerve roots.

**Conclusion:**

sc-rAAVl mediates stable IT transgene expression for ≥ 3 mo. Our findings support the underlying hypothesis that IT target cells for gene transfer lack the machinery for efficient conversion of the single-stranded rAAV genome into double-stranded DNA and favor uptake of serotype 1 vectors over 2. Experiments presented here will provide a rational basis for utilizing IT rAAV gene transfer in basic and translational studies on chronic pain.

## Background

The spinal cord (SC) is an important center of nociceptive signal processing and a notable anatomic target for analgesic therapy development. Delivery of drugs to the SC by common systemic routes, i.e. pills taken by mouth, patches applied to the skin, and injections given intravenously has two potential pitfalls: Firstly, the drug may not cross the blood brain barrier (BBB) if it is hydrophilic and/or has a high molecular weight. Secondly, once across the BBB, it may cause untoward effects by acting indiscriminately on supraspinal structures of the central nervous system (CNS). Intrathecal (IT) delivery addresses these issues by targeting drugs anatomically to the SC. While IT pharmacokinetics may vary depending on the drug's lipophilicity, its stability in the cerebrospinal fluid (CSF), the flow pattern of CSF in an individual animal or patient, and other modifying factors [1], this form of administration has in practice been found to allow specific spinal targeting of a variety of agents. It has been used in laboratory animals for decades [2,3] and proven to be efficacious in patients [4]. However, the need to implant and maintain an IT catheter and to infuse drugs through a pump system to achieve a long-term effect requires a specialized medical team [5] and can be complication-ridden [6-8]. This has effectively prevented the adoption of IT pain therapy in large groups of patients who could benefit, e.g. many with advanced cancer under the primary care of oncologists. In the investigational setting, targeting of a newly identified spinal pain mechanism may be curbed by the lack of an appropriate small molecule or recombinant protein. In many such instances the corresponding cDNAs are available and expressing them IT would suffice to proceed with a particular experiment or a translational clinical study.

IT gene transfer has been investigated by several groups as an alternative to IT peptide or protein delivery [9-14]. The cells reached with current gene vectors in the IT space, e.g. meningeal fibroblasts, are not directly involved in nociceptive signal processing but can assume an active role by secreting a relevant peptide or protein (encoded by the gene vector) into the CSF. The peptide or protein can then act in a paracrine fashion on cells in the spinal cord as shown in IT gene transfer experiments for β-endorphin and interleukin (IL)-10 [10-12]. Different types of gene vectors have been used in the IT space: liposomes [15], plasmid DNA with electroporation [13,14], plasmid DNA in hypertonic diluent [16], adenovirus [9-11], and adeno-associated virus (AAV) [12]. While all studies reported some promising evidence of transgene activity, the duration of expression never exceeded two weeks (except for a protocol using repeat vector administration [16]). Thus, long-term gene expression has not been achieved with IT delivered vectors. For other body sites, the problem of limited duration of transgene expression has been a common finding and a favorite topic of gene therapy research. Few vector systems have emerged that can often overcome this problem, in particular vectors derived from AAV, lentiviruses, or herpes simplex viruses (HSV). Recombinant AAV (rAAV) is popular because it may be a particularly safe choice. It is derived from the adeno-associated virus that commonly infects humans without ever causing symptoms or a disease. rAAV vectors are engineered to be devoid of viral genes, integrate rarely into chromosomes, and appear to pose little risk to the genomic integrity and growth control mechanisms of primary cells. Clinical safety and efficacy data on rAAV is currently accumulating from several clinical trials for retinal degeneration, hemophilia, Parkinson's, and other diseases [17].

The overall goal of the present study was to develop IT gene transfer yielding true long-term expression. We defined this as stable expression for at least three months following a single vector administration. Many chronic pain models, e.g. the chronic constriction injury model of neuropathic pain induce alterations in nociceptive behaviour for approximately two months thereby exceeding the two-week limit of previous reports on IT gene transfer but staying within the time frame set here. We defined "IT" administration as atraumatic injection into the lumbar CSF, i.e. modelling a lumbar puncture (LP). A LP is a clinically safe procedure because it does not sever the SC. It thereby differs from reports "subarachnoid" vector delivery achieved by a laminectomy at a higher SC level followed by blind transdural vector injection with a sharp pipette [18], which leads to highly localized gene expression consistent an intraparenchymal injection into the SC [19]. We focused on AAV based vectors even though a recent study in this journal had reported that IT rAAV2 expressed only for two weeks [12]. We hypothesized that the IT performance of the conventional single-stranded rAAV serotype 2 used in the published studies was limited by a failure of the capsid protein to mediate uptake and intracellular virus particle processing and/or by a failure of the cellular machinery to mediate second-strand DNA synthesis, which is a requirement for gene expression from a single-stranded DNA parvovirus like AAV. We tested these possibilities using rAAV vectors pseudotyped with capsid proteins of the serotypes 1, 2, 3, and 5 and by employing a recently developed rAAV variant that is packaged as double-stranded DNA, a "self-complementary" rAAV (sc-rAAV). By combining both approaches, we found that sc-rAAV serotype 1 has superior IT gene transfer capability mediating reliable and stable IT gene expression for at least four months in rats.

## Results

### Modification of rAAV capsid and ITR for improved IT gene transfer

rAAV2 is the best characterized type of rAAV vector and has been reported to mediate long-term gene expression in the brain, liver, muscle and other organs [20]. We prepared rAAV2 expressing enhanced green fluorescent protein (EGFP) under the control of the CMV promoter and injected it IT into twenty rats at doses up to 10^10 ^viral particles per animal. Ten control animals were injected with phosphate buffered saline (PBS). Animals were sacrificed after four weeks or four months, because rAAV2 has been reported to reach maximal expression levels in this time frame in other organs [21]. SC tissue from half of the animals was prepared for Western blotting (fresh frozen at -80°C) and from the other half for microscopy (requiring formalin fixation). No EGFP could be detected by Western blotting of tissue pooled from the upper and lower half of the SC or by extensive review of sections from all SC segments including the cauda equina (CE) using laser scanning microscopy (data not shown).

**Figure 1:**
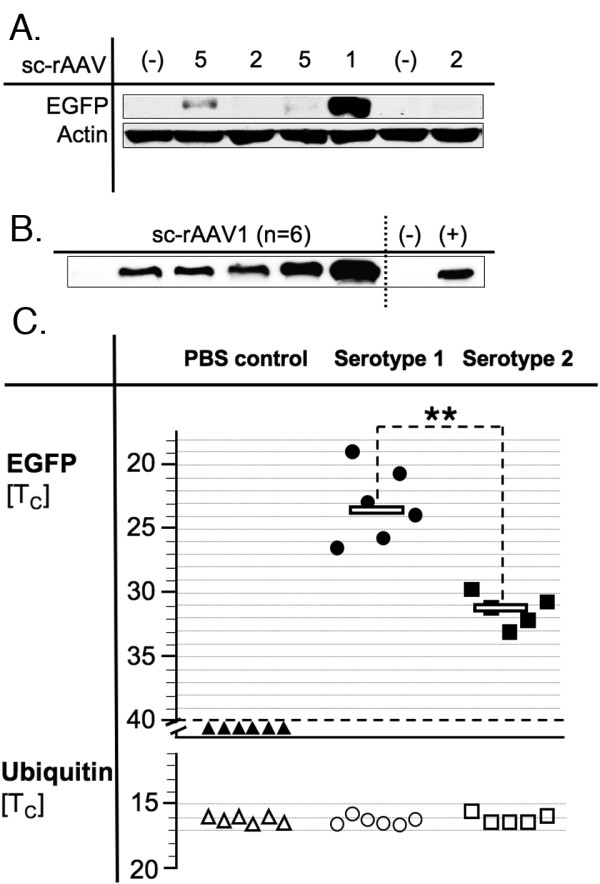
**Improved IT gene transfer by pseudotyping sc-rAAV vectors with the serotype 1 capsid**. (A.) sc-rAAV vectors packaged with capsids of the serotypes 1, 2, and 5 were evaluated. Four months after IT vector application (3 × l0^9 ^particles/animal), animals were sacrificed, the lower half of the SC including the CE and the surrounding meninges was harvested, frozen at -80°C, pulverized, and subjected to Western blotting (20 μg protein loaded per lane). A representative Western blot demonstrating EGFP expression in rats injected with vectors of the serotypes 1 and 5 is shown. No expression was seen with serotype 2 or in negative control rats injected with PBS (marked as '-'). Actin served as a loading control. (B.) One month after IT vector administration expression with serotype 1 was demonstrable in five out of six rats. The failure to detect EGFP expression in one rat may have been related to IT bleeding at the time of catheter placement and/or to inattention in harvesting the nerve roots of the CE, which were included with the lumbar segments of the SC in this experiment. '(+)' indicates expression in the SC of an EGFP-transgenic mouse, which served as positive control. Equal loading of lanes was confirmed by protein staining of the membranes (not shown). (C.) qPCR demonstrated significantly higher IT expression with serotype 1 (●) compared with serotype 2 (■) (p < 0.01). The scale of the ordinate is inverted indicating the threshold cycle T_c _with lower cycle numbers corresponding to higher expression. No expression was seen in PBS controls (▲) as indicated by T_c _> 40. The T_c _for the house keeping gene ubiquitin varied little and was not different between the groups.

Gene transfer with AAV2-derived vectors can be improved for certain applications by "pseudotyping" the recombinant vectors with capsids of other AAV serotypes while retaining the AAV2-derived inverted terminal repeats (ITR) and other elements of the viral genome [22,23]. The resulting vectors are designated rAAV2/l, rAAV2/2, rAAV2/3 and so on indicating the capsid serotype. We tested rAAV2/l and rAAV2/5 vectors IT but were unable to detect green fluorescent protein expression by laser scanning fluorescent microscopy. Repeating the experiment with rAAV2/l and rAAV2/5 vectors expressing canine factor IX (a generous gift from Dr. H. Chao) and measuring transgene levels in the CSF by ELISA did not provide any evidence of transgene expression (data not shown). We reasoned that the target tissues that are accessible to gene vectors in the IT space might lack cellular factors required for second-strand DNA synthesis. AAV is a single-stranded DNA parvovirus and synthesis of a complementary DNA strand is an absolute requirement in the life-cycle of wild-type AAV and for expression of genes encoded by any recombinant variety [24]. To test if getting around this step would facilitate IT gene expression, we prepared self-complementary rAAV (sc-rAAV) vectors that contain a deletion in the D-region of one of the ITRs. During viral replication, this deletion prevents nicking of the newly synthesized rAAV genome by the viral protein rep thereby allowing efficient production and packaging of dimeric, double-stranded rAAV genomes into the recombinant viral particles [25]. We prepared sc-vectors of four different serotypes, sc-rAAV2/l, sc-rAAV2/2, sc-rAAV2/3, and sc-rAAV2/5, and tested them in small groups of animals using 3 × l0^9 ^sc-rAAV particles per IT injection as for the prior experiments. Animals were sacrificed after four months and the lower half of the SC (lumbar segments and cauda equina) was analyzed by Western blot. sc-rAAV2/l resulted in strong EGFP expression and sc-rAAV2/5 in weak expression. No expression was seen with sc-rAAV2/2 or sc-rAAV2/3 (not shown) or in PBS injected control animals as illustrated in a representative Western blot (figure 1A). To test if expression could be detected at an earlier time point, we sacrificed animals one month after IT sc-rAAV2/l injection and found strong EGFP expression in the majority of SC samples. Expression levels were in the range of 1% of EGFP-transgenic mice used as a positive control in this part of the study (figure 1B). To confirm that the improved outcome with sc-rAAV2/l over sc-rAAV2/2 was indeed related to the serotype, we repeated the experiments in two new groups of animals with newly prepared vectors. We used quantitative reverse transcriptase polymerase chain reaction (qPCR) to quantitate low levels of sc-rAAV2/2 expression that had not been detectable by Western blot. As shown in figure 1C, qPCR of the house keeping gene ubiquitin showed little inter-animal variation and no difference between groups. qPCR from control SC from PBS injected animals did not reach the detection threshold (at the maximum of 40 cycles). Expression with serotype 1 was significantly higher than with serotype 2 (p < 0.01) confirming the prior finding.

### Transgene expression in the brain after IT vector delivery

**Figure 2:**
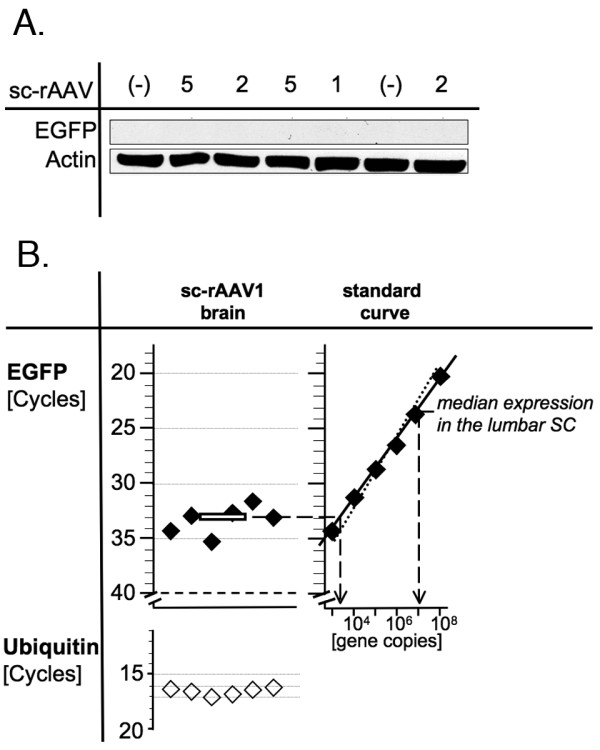
**Low levels of transgene expression in the brain following IT sc-rAAV delivery**. (A.) EGFP expression in the brain was undetectable by Western blotting. Rats were injected IT with sc-rAAV vectors of different serotypes as indicated (3 × l0^9 ^particles/animal). (B.) Low levels of EGFP expression could be detected in the brain by qPCR (sc-rAAV2/l, 3 × l0^9 ^particles/animal). The standard curve demonstrates that the median T_c _for EGFP expression in the brain corresponded to only 2 × 10^-4^-fold the expression found in the lower SC.

One of the goals of IT gene delivery is to minimize exposure of the forebrain to the effects of the vector-encoded transgene. In our study, no EGFP expression could be detected in the brain by Western blot (figure 2A). Based on experiments with recombinant EGFP protein, we estimated that the sensitivity of the Western blot was approximately 2–20 pg/sample under optimized conditions and would allow us to detect levels in the brain that are approximately 10^-2^-fold the average level in the cauda equina. In order to be able to detect lower levels and to make quantitative comparisons over a wider range, we used the qPCR introduced in the prior figure establishing a standard curve demonstrating linearity over a 10^5^-fold range. Low levels of EGFP were detected in the brain, which were 2 × 10^-4^-fold the levels detected in the lumbar third of the SC (figure 2B). Of note, we used the lumbar portion of the SC in this experiment rather than the CE, because we found it difficult to isolate sufficient quantities of high-quality RNA from the CE. As described in the next section, expression is highest in the CE (as assessed by Western blotting) suggesting that there is a greater than 10^5^-fold difference in expression levels between the lower IT space and the forebrain.

### Anatomical localization of transgene expression

**Figure 3:**
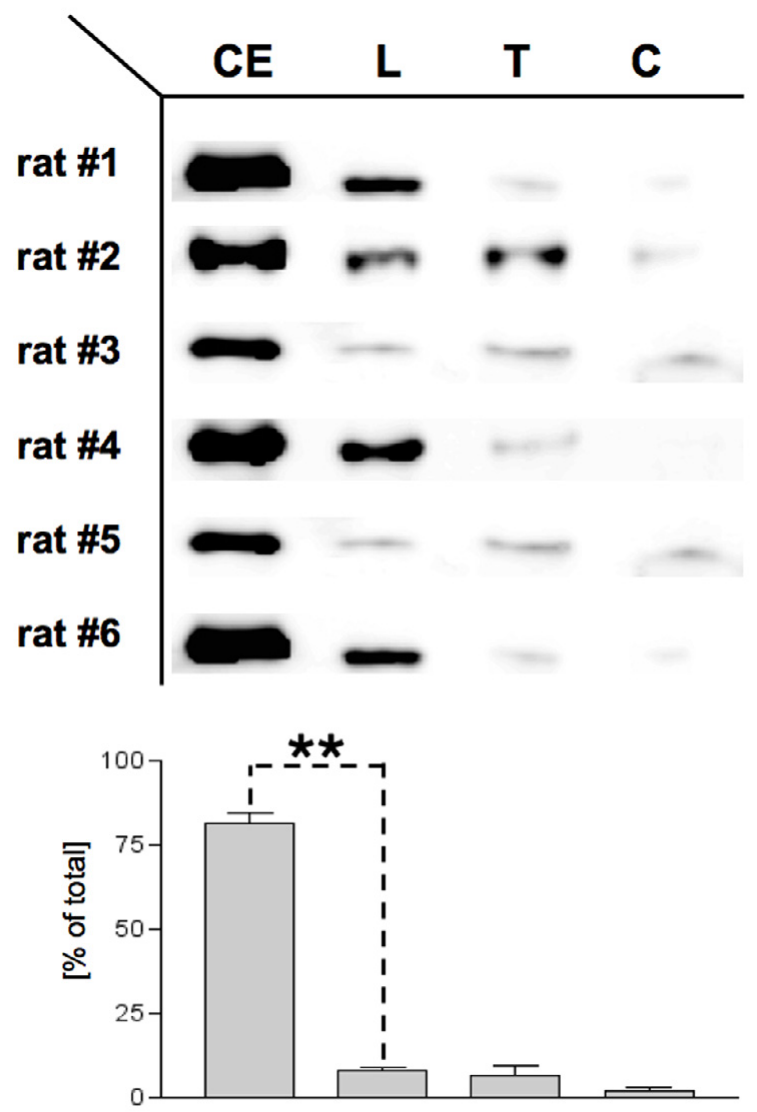
**Anatomic distribution of transgene expression along the spinal axis**. EGFP expression was consistently higher in the cauda equina (CE) than in the rostral parts of the SC. The CE and SC were harvested as one tissue block with the meninges intact, dissected into four portions, CE, lumbar SC ("L"), thoracic SC ("T"), and cervical SC ("C") and subjected separately to Western blotting. Quantitation of expression levels demonstrated a significant difference between the CE and the adjacent lumbar SC (p < 0.01).

Quantitative Western blotting demonstrated that EGFP expression was highest in the CE followed by the lower and then the upper sections of the SC forming an apparent gradient from the site of vector delivery in the area of the conus medularis to the rostral end of the SC (figure 3A).

We next localized EGFP expression in microscopic sections of the SC by laser-scanning microscopy. Using SC tissue from EGFP-transgenic and control mice as positive and negative controls, we established settings that suppressed background fluorescence and/or identified areas of unspecific fluorescence by their broader emission spectrum. Following an open label training period, the principal microscopist in the team (NMH) was given 15 slides in a blinded fashion that had been selected randomly from a collection of two hundred slides by a person not involved in the study and not knowledgeable of the animal's group assignment. Slides included sections from all parts of the SC and CE and were in roughly equal proportion from animals injected either with sc-rAAV2/l or with PBS. Examination of the slides under these conditions led to the correct identification of 7 of 7 tissue samples as positive, i.e. prepared from rats injected IT with sc-rAAV2/l and of identification of 8 of 8 samples as negative, i.e. prepared from rats injected with PBS. If the outcome of such an experiments is determined by chance, it follows a binomial distribution since the outcome is binary (correct/incorrect), the experiment consists of independent trials (each slide was evaluated independently of the outcome for the previous slide), the number of trials is fixed in advance, and an equal probability of success for each individual trial (each slide) exists, which is similar to the flip of a fair coin = 0.5. Thus, we calculated that the likelihood of 15 consecutive successes (= correct identification of slides) was (l/2)^15 ^= 6.1 × 10^-5^, which means that the result is highly significant (p < 0.0001) and unlikely to consequence of chance. This argument provided us with confidence that the fluorescence observed under these conditions reflected a true gene transfer effect. The majority of specific fluorescence was observed in the nerve roots. A typical example from the lumbar SC is shown in figure 3B. This finding is consistent with the Western blot finding of figure 3A, namely that the portion of the SC with the greatest density of nerve roots, the CE, expresses EGFP at the highest level. We cannot exclude EGFP expression in other areas including possibly the DRG. In fact, on occasion we had the impression that EGFP could also be observed in meningeal fibroblasts and occasionally in cells intrinsic to the SC. These possibilities are currently under investigation with higher titer vectors preparations and improved detection techniques based on immunohistochemistry.

### Long-term gene expression

**Figure 4:**
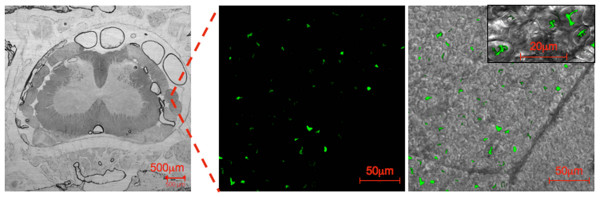
**Cellular localization of transgene expression**. Confocal microscopy demonstrated EGFP-expression in the nerve roots. The left panel provides an anatomic overview of a lumbar section through the spinal cord obtained with transmission light at a low magnification. The middle panel shows EGFP fluorescence (excitation wave length 488 nm, emission wave length 505–530 nm). The right image is a fusion of the fluorescence image with a transmission light micrograph of the same area. The insert depicts a detail at higher magnification. Images were obtained on an LSM 510 META confocal microscope.

The major objective of this study was to identify a vector for IT gene transfer capable of long-term gene expression, i.e. beyond the two-week time point reported by all previous studies on IT gene transfer. As shown in figure 1A, we initially identified sc-rAAV2/l as a good candidate vector based on screening experiments employing a four months-time point. To formalize this finding we determined the level of EGFP expression by quantitative Western blotting of CE tissue at several time points after IT administration of sc-rAAV2/l. As shown in figure 4, significant expression was detectable one week after vector delivery followed by a further increase. Gene expression then reached a plateau by four weeks and persisted unchanged at that level for at least twelve weeks. This time course, i.e. a slow onset of gene expression over the course of several days to a few weeks followed by persistence of gene expression for many months is typical of successful AAV gene transfer experiments in other organs. Self-complementary AAV vectors as used in this study have been reported to have a faster onset of gene expression than conventional, single-stranded AAV vectors. Therefore, we repeated the first two time points in a new group of animals. Again, we found that expression at one week was lower than expression at four weeks.

## Discussion

The present study addressed the question if rAAV vectors constitute a notable improvement upon other gene vectors currently used for IT rat studies on chronic pain, in particular, if rAAV vectors can mediate efficient long-term gene expression in this paradigm. To overcome known inadequacies of conventional rAAV2 in the IT space, we tested alterations to its two principal components, the capsid and the genome. Specifically, we pseudotyped rAAV vectors with capsids from various serotypes and modified its genome to allow high-efficiency packaging of double-stranded (self-complementary, "sc-") instead of single-stranded recombinant genomes. While the parent vector, rAAV2 performed poorly yielding low expression levels that were detectable only by qPCR, a vector carrying both above modifications, sc-rAAV2/l performed favourably. EGFP marker gene expression was reproducibly detected *in vivo *by qPCR and Western blotting and under blinded conditions by confocal microscopy. Transgene levels were found by Western to be highest in the CE and the caudal SC, which was consistent with our microscopic observations that the nerve roots displayed strong specific EGFP fluorescence. Expression occurred as early as one week, increased subsequently, and remained stable between four and twelve weeks. These results supported the underlying hypothesis that the poor performance of rAAV2 vectors resulted from an inability of potential target cells in the rat IT space to mediate binding of serotype 2 capsids and/or to provide the machinery for efficient conversion of the single-stranded vector genome into a double-stranded DNA template ready to be transcribed. While further questions regarding the IT properties of these vectors could be addressed with additional marker gene studies as discussed below, the presented data should allow the efficient design of improved vectors for IT delivery in chronic pain models, e.g. since completion of the study we were able to construct an sc-rAAV2/l vector expressing a secreted analgesic gene product that we could detect in CSF from the lumbar IT space and the *cisterna magna *two months after IT vector application (data not shown).

The utility of gene therapy for a specific medical application depends critically on how well the vector properties match the requirements set forth by the molecular target. IT gene therapy for pain was proposed a decade ago in a report on an artificial opioid gene with paracrine spinal analgesic activity [26]. When the gene was delivered IT by an adenovirus vector [10] or by grafting of retrovirally transduced fibroblasts [27] it suppressed nociceptive behaviour in rats. However, the approach had shortcomings that discouraged further exploration in pre-clinical studies. Firstly, it did not allow for regulation of the therapeutic gene following vector delivery, which may have been desirable in this case, because opioid medications typically require dose adjustments in the clinical setting. Secondly, gene expression and thus analgesic activity was not sustained beyond two weeks due to the gene transfer technology, namely an inflammatory response in the case of the adenoviral vectors and promoter shut-down in the case of the Moloney-type retroviral vectors. In the early era of gene therapy research cases like this – an appealing idea, support through a "proof of principle" study, then the realization that no available vector could do the job well enough – were common precipitating a call to pursue "The basic science of gene therapy" [28], i.e. a focus on studying the vectors in order to match their biological properties to a potential application or to improve their shortcomings. In the area of gene therapy for pain Drs. Glorioso, Fink, Yeomans et al. took the former approach by building their pre-pro-enkephalin gene transfer around the natural ability of HSV to infect peripheral sensory nerves and be retrogradely transported to the DRG [29-31]. Others including ourselves pursued the latter approach, i.e. searching for an improved vector system to achieve better gene delivery by the administration route that appears most appropriate for targeting spinal pain mechanisms, namely IT delivery. In a recent review article we provided an account of which vector types were tested by ourselves and others for IT use failing to provide sustained expression, why IT AAV appeared promising despite the limitations encountered in published studies, and which molecular targets characterized by others may be most suitable for IT therapy of chronic pain states without requiring regulation of the therapeutic gene or dose adjustments except through the delivered dose of vector [32]. In the present article, we show the result of our search for a better vector providing the first report of long-term gene expression after a single IT administration of any vector type. The approach should be useful in its present form for studies in rats requiring chronic delivery of a secreted transgene product into the spinal CSF, e.g. IL-10 for the suppression of glial activation as proposed by Drs. Milligan, Watkins et al. [11,12,33,34]. The AAV vector dose used in this study, 3 × l0^9 ^viral particles per animal could be titrated downwards if lower transgene levels are desired that are in line with levels obtained with naked DNA/plasmid gene transfer (unpublished). AAV vector doses can also be increased to ≥ 10^11 ^viral particles per rat with well purified/concentrated AAV preparations while keeping the IT injection volume constant. This should provide flexibility for different therapeutic genes and experimental goals.

Extrapolating from the experience with rAAV delivery to other body sites (muscle, liver, brain), it can be expected that IT delivered rAAV does not induce cellular immunity to vector component in rodents and will have minimal or no effects ascribable to the gene transfer procedure thereby permitting a clean evaluation of the transgene of interest in mice or rats. rAAV appears to be among the safest gene transfer options for potential use in patients and is currently undergoing clinical evaluation for retinal degeneration, hemophilia, Parkinson's and other diseases [17]. It may therefore be a candidate vector for future translational studies serving as a research tool to validate whether the manipulation of a specific spinal molecular mechanism that is operative in rodent models is similarly important in patients, e.g. glial activation in neuropathic pain. In order to evaluate rAAV for such use, a number of hypotheses need to be tested in large animals, which are related to the vector technology. Up-scaling experiments to large animals has been a critical benchmarks in other fields of gene therapy research, e.g. hemophilia, an area in which one of us (CEW) has been closely involved with this issue [35,36]. Establishing in pigs, dogs, or non-human primates whether a serological and/or cellular immune response occurs to IT rAAV and, perhaps more importantly, to a specific transgene product of interest will be critical [37]. In addition such experiments would allow to determine gene product levels, biodistribution, and target cells of IT rAAV in a model that appears more realistic for humans than rodents. Hopefully, experiments as presented and proposed here will provide a rational basis for utilizing gene transfer in basic and translational studies on chronic pain.

## Methods

### Animals and vector application through an IT catheter

All procedures involving animals were reviewed and approved by the Institutional Animal Care and Use Committee (IACUC) and followed national guidelines and requirements set forth by the National Institutes of Health (NIH), Bethesda, MD, USA. Adult male Sprague-Dawley rats of 300–350 g body weight were used.

Intrathecal catheterization was performed as originally developed by Yaksh et al. and described in a recent review on IT catheterization and drug delivery in the rat [3,38]. In brief, rats were anesthetized with isoflurane and placed in a stereotactic frame. The occipital neck area was shaved and sterilized with betadine solution (Purdue Fredrick, Stamford, CT, USA). A skin incision of approximately 1 cm length was made and the atlanto-occipital membrane was surgically exposed using blunt preparation technique with meticulous attention to hemostasis. A l-2 mm long incision was made in the atlanto-occipital membrane cutting the dura and arachnoid membrane and exposing the subarachnoid space. Next sterile catheter consisting of slightly pre-stretched PE10 polyethylene tubing (Intramedic, Becton Dickinson and Company, cat. no. 427400) was inserted through the *cisterna magna *into the IT space and advanced to the caudal end of the SC. rAAV particles suspended in phosphate buffered saline (PBS) was then injected in a total volume of 15 μl at constant flow rate over one min (minute). An equal volume of PBS without virus was injected in control animals. 3 × l0^9 ^viral particles were used for all experiments except for some of the rats used for confocal microscopy, which were injected with 1× or 3 × l0^10 ^viral particles. The catheter was then slowly withdrawn, the neck muscles approximated with surgical sutures and the skin closed with 9 mm wound clips (Clay Adams Brand, Becton Dickinson, no. 427631). Animals were placed in a recovery cage and monitored until they resumed normal activity. Animals were examined postoperatively and daily thereafter for signs of neurological deficits or any kind of distress and animals found to display any such signs were sacrificed in accordance with the IACUC approved procedures.

### Quantification of gene expression by Western blotting and reverse transcriptase PCR

Animals were sacrificed by carbon dioxide inhalation. The SC and CE were immediately removed with the meningeal linings intact and snap frozen on a metal block cooled I liquid nitrogen. The SC/CE tissue block was then subdivided into two or four portions along the rostral-caudal axis as indicated in the respective figure legend and pulverized in a ceramic grinder under liquid nitrogen.

For quantification of EGFP protein by Western blotting, an aliquot of the pulverized tissue was homogenized in RIPA buffer on ice with a high speed tissue homogenizer (Tissuemizer, Fisher Scientific) and centrifuged for 30 min at 13,000 rpm at 4°C allowing elimination of cell debris found in the pellet. The protein concentration of the supernatant was determined with the DC Protein Assay Kit II according to the manufacture's instruction (Bio-Rad). Spinal cord tissue of an EGFP transgenic mouse or recombinant EGFP protein (Clontech) was used as a positive control and quantitative standard. 20 ug total protein was electrophoresed in a 12% SDS-polyacrylamide gel and electroblotted onto a nitrocellulose membrane. The membranes were incubated in blocking solution containing 5% nonfat milk in 1× Tween Tris-buffered saline. Membranes were then incubated with the mouse monoclonal antibody against EGFP (JL-8, BD-Biosciences, dilution 1:1000) for 2 h at room temperature. Membranes were washed and incubated anti-mouse horse-reddish peroxidase-conjugated antibody (Amersham, dilution 1:2500) for 1 h at room temperature. Membranes were again washed and then covered with the Lumi-Light Western Blotting Substrate (Roche). The resulting chemiluminescence was then digitally recorded using the a cooled camera luminescent imaging system (LAS-3000, Fuji).

For quantification of EGFP RNA, an aliquot of the pulverized frozen tissue was dissolved and thereby thawed in RLT buffer followed by isolation of total RNA using a mini-column based kit (RNeasy Mini Kit, Qiagen). Single-stranded cDNA was synthesized in a 25 μl reaction from 1 μg total RNA using 2.5 ul 10× buffer, 10 ul MgC12, 2.5 ul dNTP, lul oligo dT, 0.5 ul Rnasin, 0.8 μl RT, and nuclease free water at 25°C for 10 min, and 45°C for 60 min followed by inactivation at 95°C for 5 min (Reserve Transcription Kit, Promega).

Quantitative PCR was performed in triplicates on an ABI PRISM 7900HT thermocycler (Applied Biosystems) using the fluorophore SYBR Green (Applied Biosystems). A cDNA amount corresponding to 15 ng of RNA was used per reaction in a total volume of 10 μl. The concentration of each primer was 0.4 μM. Primers for EGFP were: Forward 5'-AGC AAA GAC CCC AAC GAG AA-3', reverse 5'-GGC GGC GGT CAC GAA-3'. Primers for rat ubiquitin were: Forward 5'-GTG GCT ATT AAT TCT TCA GTC TGC AT-3', reverse 5'-GCA AAT GGC TAG AGT GCA GAG TAA-3'. Following incubation at 50°C for 2 min and denaturation at 95°C for 10 min 40 thermal cycles were executed as follows: 95°C × 15s, 60°C × 1 min.

### Tissue slide preparation and confocal microscopy

Animals were anesthetized with Chloral Hydrate 30% (Sigma, Lot no. 045K0664) dissolved in sterile water and injected intraperitoneally at a volume of 0.8–1.4 mL, which was repeated if needed to achieve deep anesthesia. Animals were then perfused transcardially for 1 min with freshly prepared ice-cold paraformaldehyde 1% (PFA, Electron Microscopy Sciences, catalogue number 19210) in PBS, followed by perfusion with PFA 4% and glutaraldehyde 0.125% (Electron Microscopy Sciences, 50% solution, cat. no. 16310) for 13 min. The spinal column was then removed by first exposing it in its entire length from the cisternal membrane to 14 cm caudally by anatomical dissection of the dorsal muscles and by then transecting of the ribs and other adjacent tissue. The spinal column with the SC, CE and meninges intact was then post-fixed in PFA 4% for 24 h. The spinal column was then subjected to a decalcification protocol consisting of slow-motion shaking of the tissue at 4°C in a buffer of 10% ethylene diamine tetra acetic acid (EDTA, Sigma, cat. no. E5134) prepared in distilled water and adjusted to a pH of approximately 7.4–7.6. The EDTA buffer was changed daily during the first two weeks followed by changes every other day until the bone of the spinal column was completely decalcified at 4 weeks. The tissue was then cryoprotected by immersion in increasing concentrations of sucrose (Sigma, Lot no. 62H0607) dissolved in PBS, namely at concentrations of 10% × 2 h, 20% × 2 h, and 30% overnight. The tissue was the dissected in smaller blocks, embedded in Optimal Cutting Temperature Compound (OCT, Tissue-Tek, 4583, Sukura) or Tissue Freezing Medium (TBS, Electron Microscopy Sciences, cat. no. 72592). Tissue sections of 50–60 microns were then cut on a Cryostat (2800 Frigocut E, Reichert-Jung), thaw-mounted on pre-treated glass slides (50 × 75 mm slides, Brain Research Laboratory), gently PBS-washed, and protected with a cover glass (48 × 60 mm no.l, cat. no. 4860-1, Brain Research Laboratory) using mounting medium (Vectashield hardset H-1400, Vector Laboratories).

Microscopic examination of tissue slides was performed with a confocal laser-scanning microscope (LSM 510 META, Zeiss Inc.). An excitation wavelength of 488 nm was used (Argon laser, intensity 5%). To detect EGFP, fluorescence images were recorded at an emission wavelength of 505–530 nm. Unspecific fluorescence was visualized at >560 nm. Settings for gain and offset were optimized using positive and negative control tissue and open label samples. Subsequently all settings were kept constant for the blinded examinations of slides.

### rAAV production

All rAAV vectors were produced, purified, and titered as previously described [35]. The sc-rAAV/EGFP DNA templates utilized for encapsidation in AAV 1, 2, and 5 capsids were derived from plasmid pHpa-trs-SK developed by one of the co-authors [25]. The AAV1 and AAV5 packaging plasmids were generously supplied through the Gene Therapy Center, University of North Carolina, Chapel Hill (Dr. Jude Samulski).

### Statistical methods

The significance level for the scoring of slides by confocal microscopy under blinded conditions was calculated based on the binomial distribution, since the data type in this case was categorical (success/failure = correct/incorrect scoring of a slide), trials were independent (each slide was evaluated independently of the outcome for the previous slide), the number of trials was fixed in advance (15 coded slides handed to the microscopist), and the probability of success was uniformly 0.5 for each individual trial. If all trials n are successful as in our case, the significance level is calculated in a simplified way as (probability of success)^n^.

The data of all other experiments was continuous (i.e. non-categorical), unpaired, and of an unknown distribution type. Therefore a two-sided, non-paired Wilcoxon-Mann-Whitney test was used to assess differences between groups.

## List of abbreviations

AAV: adeno-associated virus

BBB: blood brain barrier

CE: cauda equina

CNS: central nervous system

EGFP: enhanced green fluorescent protein

HSV: herpes simplex viruses

IACUC: Institutional Animal Care and Use Committee

IT: intrathecal

ITR: inverted terminal repeats

min: minute

PBS: phosphate buffered saline

qPCR: quantitative polymerase chain reaction

rAAV: recombinant adeno-associated virus

SC: spinal cord

sc-rAAV: self-complementary recombinant adeno-associated virus

sc-rAAV2/l: self-complementary recombinant adeno-associated virus type 2 pseudotyped with a capsid from serotype 1 (= packed in capsid protein from AAV1)

## Competing interests

The author(s) declare that they have no competing interests.

## Authors' contributions

Benjamin Storek assisted in IT vector application, harvested all tissue and performed all transgene quantifications by Western blotting and qPCR shown in figure 1C, 2B, 3A, and 4. He performed data entry for these experiments, and assisted in the analysis and preparation of figures. He also assisted in the writing of the respective Methods sections.

Nina M. Harder performed the IT vector application for figure 1C, 2B, 3, and 4B. She performed transcardial perfusions, decalcified spinal column specimens, prepared tissue slides, and performed the blinded analyses by confocal microscopy described in the Results section and prepared the micrographs for figure 3B.

Michaela S. Banck provided technical advice in the pilot stage of the study, contributed to the experimental work for figure 1A, 1B, and 2A, provided suggestions and guidance throughout, and reviewed and revised the manuscript.

Cheng Wang assisted in the production and characterization of rAAV vectors at Mount Sinai School of Medicine.

Douglas M. McCarty provided AAV plasmid constructs for production of sc-vectors prior to their publication and oversaw vector production by the core facility of the Gene Therapy Center, University of North Carolina, Chapel Hill.

William G.M. Janssen instructed NMH in transcardial rat perfusion and the preparation of microscopic slides and provided guidance and advice in optimizing tissue preparation after decalcification.

John H. Morrison provided guidance and advice throughout, donated critical resources during the initiation period (temporary laboratory space for BS and ASB) and allowed access to specialized equipment throughout.

Christopher E. Walsh provided guidance and general advice in the preparation, manipulation, and use of AAV vectors throughout.

Andreas S. Beutler initiated and oversaw all stages of the study. He carried out or actively participated in experiments for figure 1, 2, and 4 and supervised the experimental procedures for figure 3. He carried out all analyses, wrote the manuscript, and takes primary responsibility for the integrity of the data and the main conclusions.

**Figure 5:**
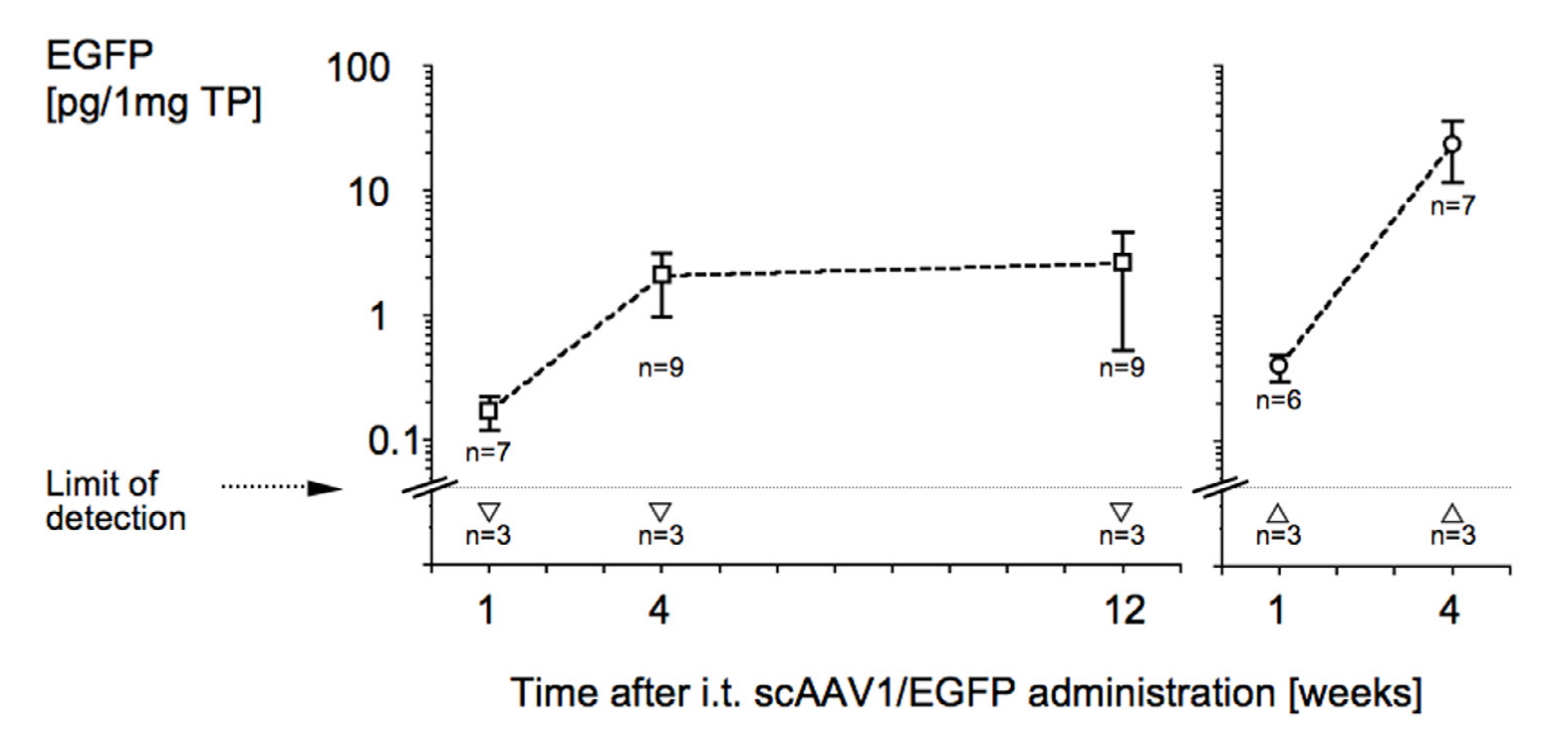
**Time course of IT gene expression**. (A.) Gene expression was detectable in the CE one week after IT delivery of sc-rAAV2/l (3 × l0^9 ^particles/animal). Expression levels increased further after one week and reached a plateau with stable expression at four and twelve weeks, the longest time point tested. Shown is the mean and SEM of expression levels determined by quantitative Western blotting with group sizes of seven to nine animals as indicated (□). No expression was seen in PBS injected control rats (▽). (B.) The one- and four-week time points were repeated in a second group of animals to confirm the rise in EGFP levels during this time frame (○). PBS controls showed again no detectable expression (△).
